# Evaluation of Mir-1290 as a Possible Diagnostic Factor in the Serum of Oral Squamous Cell Carcinoma Patients with Qualitative Real-Time Polymerase Chain Reaction

**DOI:** 10.30699/IJP.2024.2010130.3161

**Published:** 2024-02-15

**Authors:** Siavash Bagheri Shirvan, Mehdi Shahabinejad, Farnaz Mohajertehran, Nazanin Nazari

**Affiliations:** 1 *Student Research Committee, Faculty of Dentistry, Mashhad University of Medical Sciences, Mashhad, Iran*; 2 *Oral and Maxillofacial Pathology Department, Oral and Maxillofacial Diseases Research Center, Faculty of Dentistry, Mashhad University of Medical Sciences* *, Mashhad, Iran*; 3 *Oral and Maxillofacial Diseases Research Center, Mashhad University of Medical Sciences, Mashhad, Iran*

**Keywords:** Biomarker, miR-1290, Oral squamous cell carcinoma, Quantitative real-time polymerase chain reaction

## Abstract

**Background & Objective:**

This study aimed to determine the incidence of microRNA (miRNA; miR-1290) in the serum of oral squamous cell carcinoma (OSCC) patients compared to a control group using the qualitative real-time polymerase chain reaction (PCR) method.

**Methods:**

Blood serum samples were obtained from patients diagnosed with OSCC and confirmed through biopsy. The samples were collected from patients referred to the Mashhad Dental Faculty and Ghaem Hospital. The OSCC group consisted of 17 patients, while the healthy group included 15 individuals. RNA was extracted from the patient samples, and samples with an A260/280 ratio between 1.8 and 2.0 (indicating acceptable RNA quality) were immediately converted into complementary DNA (cDNA) using albumin and cDNA reference genes. The SYBR green real-time reverse transcriptase PCR method was used to measure the presence of miR-1290 in the blood samples.

**Results:**

A total of 32 patients were examined in this study, including 17 women (53.1%) and 15 men (46.9%). The mean age was 46.7 years in the healthy group and 54.6 years in the SCC group, indicating a significant difference (*P*<0.05). The expression level of the miR-1290 gene was higher in patients with SCC compared to the healthy group (*P*=0.000). While the expression level of miR-1290 was higher in grade 3 and advanced stage than in grades 2 and 1 and early stage, the differences were not statistically significant (*P*=0.173 and *P*=0.564 for grade and stage, respectively).

**Conclusion:**

The expression level of miR-1290 may increase in SCC patients compared to healthy individuals, making it a potential circulating biomarker. Further investigations for diagnostic utility would be warranted.

## Introduction

Oral squamous cell carcinoma (OSCC) is the most common malignancy in the oral cavity. Despite advancements in diagnostic and treatment procedures, the 5-year survival rate of OSCC remains around 50%. Early detection and diagnosis are thus essential for better clinical outcomes (1).

MicroRNAs (miRNAs) play an important regulatory role in cancer progression. These small, noncoding RNAs, which contain 18-24 nucleotides, regulate several key processes in cancer, including cell death, proliferation, metastasis, and treatment resistance. Over 2,500 mature human miRNA sequences have been discovered to date, according to the miRBase database. It is estimated that miRNAs control the expression of around one-third of protein-coding genes at the post-transcriptional level (2, 3). By regulating biological processes (such as cell growth, proliferation, differentiation, and death), some miRNAs act as tumor suppressors, while others promote oncogenesis.

Several studies have identified circulating miRNAs in bodily fluids like serum, plasma, and cerebrospinal fluid (CSF), which may serve as diagnostic or prognostic biomarkers in OSCC patients. Specifically, tumor suppressor miR-1017, miR-1378, miR-1869, and miR-37510 have been reported to be downregulated in OSCC tissue (4-7).

Recent research has revealed important oncogenic roles for miR-1290 across multiple cancer types. MiR-1290 was identified as a tumor-initiating, cell-specific miRNA in non-small cell lung cancer (NSCLC) (8). Serum miR-1290 levels were also correlated with chemotherapy response in NSCLC patients (9). Significant upregulation of miR-1290 was found in esophageal squamous cell carcinoma (ESCC) tissue, where it induced cancer cell proliferation, migration, and invasion (9). Additionally, miR-1290 has been characterized as a novel diagnostic and prognostic biomarker in colorectal cancer (10). However, the expression and functional relevance of miR-1290 in OSCC remains to be elucidated.

MiR-1290 has been implicated as an oncogenic miRNA regulating ESCC progression by targeting NFIX (11). A study in pharyngeal SCC demonstrated that miR-1290 directly suppressed 2 tumor suppressor genes, ITPR2 and MAF (12). According to these findings, miR-1290 may play an oncogenic role in driving cellular processes in ESCC. Specifically, miR-1290 has been shown to target the transcription factor nuclear factor I/X (NFIX). Compared to matched non-cancerous esophageal tissue, miR-1290 expression was significantly elevated, while NFIX decreased in ESCC samples. This inverse expression pattern was associated with aggressive disease features and worse prognosis (13).

However, there has been limited investigation into how miR-1290 expression relates to the clinical characteristics of OSCC. Consequently, it remains unclear whether miR-1290 expression profiles reflect the malignant properties of the primary tumor and their potential clinical significance in OSCC. The present study, therefore, aimed to quantify serum miR-1290 levels in OSCC patients and analyze associations with relevant clinicopathological variables.

## Material and Methods

**Study Subjects**

Blood serum samples were collected from patients diagnosed with OSCC via biopsy between February 2021 and September 2022. The corresponding demographic information (age, sex, and social habits) was obtained from the oral and maxillofacial pathology archive department of Mashhad Dental School, Mashhad University of Medical Science. The OSCC cohort consisted of an equal number of patients with low- and high-grade tumors. Patients were eligible if they were over 18 years old with a confirmed history of OSCC and had been referred to Ghaem Hospital.

For the healthy control group, we recruited individuals with no underlying systemic or inflammatory diseases and no history of malignancy.

The present study was approved by the Mashhad University of Medical Sciences Research Council, Faculty of Dentistry (No. 991683), as well as the Ethics Committee of Mashhad University of Medical Sciences (IR.MUMS.DENTISTRY.REC.1399.162). All participants provided written informed consent.

**Serum Processing and RNA Isolation**

Blood (5 mL) was collected from each participant. The blood samples were centrifuged at 3,000 RPM for 15 min to isolate the serum. The serum was immediately stored at -80°C.

For RNA extraction, 400-500 μL of serum was mixed with 800 μL of RNXplus solution (SinaClon, Iran) and vortexed to homogenize. The mixture was incubated at room temperature for 3-4 min. Next, 250 μL of chloroform (Merck, Company) was added and manually shaken for 15 s, followed by another 3-5 min incubation at room temperature. The samples were then centrifuged at 12,000 RPM for 20 min at 4°C.

The upper aqueous layer (500 μL) was carefully extracted and transferred to a 1.5-μL microtube. For RNA precipitation, 500 μL of isopropanol (Merck Company) was added to each microtube. The tubes were gently inverted, incubated overnight at -20°C, and then centrifuged at 12,000 RPM for 45 min at 4°C.

The RNA pellets were washed with 1 mL of 80% molecular-grade ethanol and centrifuged twice at 12,000 RPM for 20 s at 4°C. The ethanol was discarded, and the pellets were air-dried at room temperature for 3-4 min. Finally, the pellets were dissolved in 20 μL of DEPC water by incubating for 5 min at room temperature.

 Quantity and purity of the the extracted miRNAs were evaluated by measuring the 260/280 nm absorbance ratio using a NanoDrop device (Thermo Scientific 2000, USA).

**MiRNA and Gene Expression Analyses by Quantitative Real-Time Polymerase Chain Reaction**

Total RNA was extracted from the samples after the purification steps. Purity and amount of extracted RNA were evaluated using NanoDrop to determine if the RNA purity level was adequate for further analysis. RT-qPCR was then performed using a SYBR Green master mix kit (Thermos Scientific, Germany) and specific primers (Table 1) to quantify the relative expression of miRNA-1290 in the samples.

Q-rt-PCR has become the gold standard for detecting and quantifying RNA targets and is increasingly used in novel clinical diagnostic assays. This method measures relative increase or decrease in miRNA-1290 gene expression compared to a reference gene and control sample.

All qPCRs were performed in duplicate in separate 20-μL wells. Each reaction mixture contained 0.5 μL of each primer (10 pM), 10 μL of the SYBR Green master mix, 7 μL of DEPC water, and 2 μL (4 ng) complementary DNA (cDNA) template. Thermo-cycling conditions started with an initial denaturation at 94°C for 10 min. This was followed by repetitive denaturation (94°C for 30 s), annealing (60°C for 30 s), and extension (72°C and 30 s) cycles, ending with a final extension at 72°C for 35 s.

**Fig. 1:**
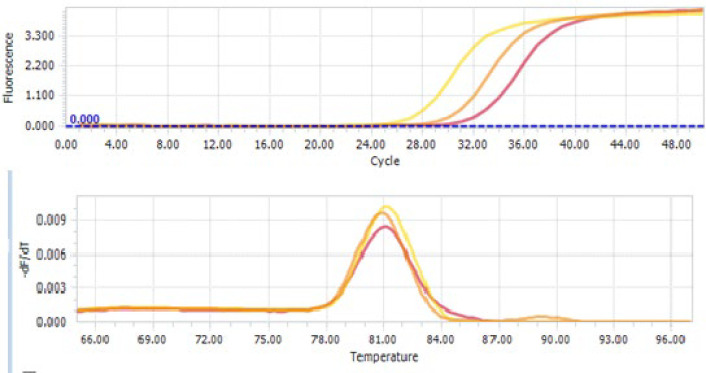
The melting curve (above) and amplification plot (bottom) of miR-1290 and GAPDH expressions in real-time PCR

Differential miRNA-1290 expression was analyzed using the ΔΔCT method, with GAPDH (NM_001289745.3) as the internal reference gene (Figure 1). A greater than 2-fold change was considered overexpression, while less than 2-fold was regarded as low or no differential expression (14, 15).

**Analytical Statistics**

SPSS version 25 (SPSS Inc., Chicago, IL., USA) was used for data analysis. The relative miRNA expression level was calculated as 2^-(ΔΔCt), a commonly used normalized measure accounting for differences in the threshold cycle number (ΔΔCt). *P*-values less than 0.05 were considered statistically significant.

**Table 1:** Specific primers for expression of mir-1290.

Primers		Sequences
Mir-1290	Forward	5’- ACA CTC CAG CTG GGT GGA TTT TTG GAT C-3
	Reverse	5’-CGG AGT CAA CGG ATT TGG TCG TAT-3
GAPDH	Forward	5’-CGG AGT CAA CGG ATT TGG TCG TAT-3
	Reverse	5’-AGC CTT CTC CAT GGT GGT GAA GAC-3

## Results

**Characteristics of the Study Subjects**

This study examined miR-1290 gene expression levels (quantitatively) in 15 control samples and 17 OSCC samples. A total of 32 patients were analyzed, comprising 17 women (53.1%) and 15 men (46.9%).

The mean age was 54.6 ± 12.85 years in the OSCC group compared to 46.7 ± 6.4 years in the control group. There was a significant difference in average age between the 2 groups (*P*=0.018). The control group consisted of 6 male patients (40%) and 9 female patients (60%). The OSCC group had 9 male patients (52.9%) and 8 female patients (47.1%).

**MiRNA-1290 Regulation in the Study Groups**

In Table 2, miRNA-1290 gene expression has been analyzed in the studied groups. MiRNA-1290 gene expression was higher in the SCC group than in the healthy group (*P*<0.001).

**Table 2:** miRNA-1290 regulation in the OSCC and control

Groups			
Group	number	mean± Standard deviation	Man-Whitney test
healthy	15	1.34±1.99	X2=-3.588 *P*=0.000
SCC	17	4.44±3.73	

**Relationship Between MiRNA-1290 Expression and Demographic Characteristics**

This study found no strong link between age and miRNA-1290 expression (ɑ=0.029 and *P*=0.912 in the OSCC cases and ɑ=0.058 and *P*=0.837 in the healthy controls). In addition, no correlation was observed between miRNA-1290 expression and gender in the 2 groups (Table 3).

**Table:** Table 3 Correlations between serum miRNA-1290 expression and gender

Group	Gender	Number (%)	MiRNA-1290 mean expression± SD	P-value
healthy	Male	6 (40)	0.8 ± 0.7	0.906
	Female	9 (60)	1.64 ± 2.54	
SCC	Male	9 (52.9)	4.83 ± 4.69	0.700
	Female	8 (47.1)	4.01 ± 2.52	

 **Relationship Between MiRNA-1290 Expression and Oral Squamous Cell Carcinoma Clinicopathological Features**

Table 4 shows that the highest expression of miRNA-1290 was related to the grade 3, but no correlation was seen between miRNA-1290 expression and the histoplogic grade. Table 4 shows an increase in miRNA-1290 expression in the advanced stage, although the difference was not statistically significant.

**Table 4:** Correlations between serum miRNA-1290 expression and the OSCC clinicopathological features

Clinicopathological Feature		Number (%)	MiRNA-1290 mean expression ± SD	P-value
Clinical Stage	Early	8 (47.1)	3.5 ± 2.64	0.56
	Advanced	9 (52.9)	5.2 ± 4.50	
Grade	I	4 (23.5)	4.1± 3.32	0.17
	II	6 (35.3)	2.5 ± 1.81	
	III	7 (41.2)	6.3 ± 4.56	

## Discussion

MiRNAs play a crucial role in regulating biological processes, and alterations in their expression can lead to the development of various human cancers (16). Specific miRNAs like miR-101, miR-137, miR-186, and miR-375 act as tumor suppressors and are downregulated in OSCC (4-6). Increased expression of oncogenic miRNAs (such as miR-497) has also been associated with metastasis in OSCC (17). Elevated miR-1290 expression has been observed across several cancer types, including colorectal (18), lung (19), ovarian (20), and acute lymphoblastic leukemia (21). Li *et al.* found that increased miR-1290 expression in pancreatic cancer was associated with decreased post-resection survival (22).

An ideal cancer biomarker, measurable in blood, body fluids, or tissues, can identify normal or abnormal physiological processes. Key attributes of a biomarker include early detection and diagnosis of malignancies, determining disease prognosis, predicting treatment response, and monitoring disease progression or risk (23).

The mechanisms by which miR-1290 is dysregulated in OSCC remain unclear. As demonstrated by Qin *et al.*, OSCC cells show decreased cyclin G2 (CCNG2) expression alongside increased miR-1290 levels (10). Downregulation of CCNG2 has also been linked to more aggressive disease and metastasis in gastric cancer (24). Moreover, miR-1290 and miR-1246 expression levels have been identified as critical factors regulating lung cancer initiation and progression (25).

In the present study, miR-1290 expression was upregulated in the OSCC patients compared to the healthy controls. Similar elevations in miR-1290 levels have been reported in tissue and serum samples from OSCC patients in studies by Qin *et al.*, Li *et al.*, and Geusau *et al.* (10, 26, 27). Sun *et al.* found that tissue miR-1290 levels correlated with serum expression and that higher miR-1290 levels were associated with better treatment response and survival in the ESCC patients (28). In contrast, Nakashima *et al.* observed a lower plasma miR-1290 expression in SCC patients vs. controls (29). It is proposed that intracellular miRNAs can be differentially secreted into blood or retained within cells depending on the disease stage (29-31). Thus, miR-1290 may remain intracellular with decreased circulating levels, specifically during early and late-stage OSCC (29).

Ideal biomarkers should indicate disease progression (23); however, in our study, miR-1290 expression was not associated with the lesion stage or grade. While Qin *et al.* reported a correlations between miR-1290 and disease stage and lymph node metastasis (10), we examined serum rather than tissue miR-1290 levels. Nakashima *et al.* found higher miR-1290 expression in more differentiated SCC samples, possibly due to their analysis of plasma vs. tissue miR-1290 (29).

A robust diagnostic biomarker should also be independent of age and gender (23). Consistent with our findings, Qin *et al.* noted no association between miR-1290 levels and gender (10). Additionally, Sun *et al.* found that miR-1290 expression did not correlate with age or gender in SCC patients (28).

This study did not compare the diagnostic utility of miR-1290 versus antigen-125, CEA, or SCC antigen biomarkers. Furthermore, conclusions regarding miR-1290 associations with grade and stage may be limited by the small sample size.

## Conclusion

This study aimed to investigate miR-1290 expression levels in the OSCC patients compared to the healthy controls. We found a significant upregulation of miR-1290 expression in the OSCC group. Although it was not statistically significant, miR-1290 levels showed an increasing trend with higher disease stages and grades. Additionally, no significant correlation were observed between miR-1290 expression and age or gender.

In summary, our findings revealed increased serum miR-1290 level in the OSCC patients vs. controls. However, larger patient cohorts are needed to conclusively determine association between circulating miR-1290 levels and the clinicopathological characteristics. Further research focusing on the functional role of miR-1290 in OSCC tumorigenesis and its potential utility as a non-invasive biomarker would be warranted.
